# Computational evaluation of the η6-arene during the ATH of imines on Noyori’s RuII catalyst

**DOI:** 10.1186/1758-2946-6-S1-P39

**Published:** 2014-03-11

**Authors:** Petr Kacer, Sot Petr, Jan Pechacek, Jakub Januscak, Marek Kuzma

**Affiliations:** 1 Institute of Chemical Technology, Prague, 166 28, Czech Republic

## 

Asymmetric hydrogenation ranks to the most intensively researched way of preparation of enantiomerically pure compounds which are demanded e.g. in pharmaceutical industry, cosmetics or agriculture. In the field of asymmetric transfer hydrogenations (ATH) of C=N and C=O double bonds Noyori’s ruthenium (II) complexes represent significant breakthrough. This catalytic system consists of three integral parts - chiral monotosylated diamine ligand – i.e. N-(p-toluenesulfonyl)-1,2-diphenylethylenediamine (TsDPEN), η^6^-coordinated aromatic molecule (e.g. benzene or p-cymene) and halogen counteranion (usually chloride). General formula of the catalyst can be written as [RuCl(η^6^-arene)(N-arylsulfonylDPEN)]. Aforementioned fragments/ligands offer countless number of possibilities for structural modifications – e.g. elongation of carbonaceous spacer between phenyl rings within 1,2-diphenylethylendiamino fragment, alkylation of amino group, usage of diversely substituted η^6^-aromatic molecule, employment of different aryls within arylsulfonyl fragment etc. Systematic evaluation of these modifications has multilateral benefits because it not only helps to clarify mechanistic phenomena but also contributes to the deeper understanding of relationship between structure and catalytic activity. With sufficiently big and rich data base it should be possible to tailor catalyst’s properties specifically for given substrate (or class of substrates) and reaction conditions (solubility, stability, etc.). Our research is focused primarily on comprehension of role of the η^6^-aromatic molecule during asymmetric transfer hydrogenation of imines. This ligand plays very important mechanistic role because its structure (respectively interaction with the substrate) allows asymmetric course of the reaction. Arene ligand can in certain cases form stabilizing CH/π interaction between aromatic part of substrate and therefore lower energy of transition state. This led us to the hypothesis that alteration of its structure could strongly affect enantioselectivity and reaction rate. This hypothesis has been brought up and discussed but only in case of ATH of C=O bonds, which dramatically differs from hydrogenation of C=N bonds. Usually only simply alkyl-substituted arene molecules are used as aromatic ligands. In our study we have prepared and compared four catalysts with different aromatic ligands (benzene, p-cymene, mesitylene, 1,2,3,4,5,6-hexamethylbenzene) according to their performance (reaction rate, enantioselectivity) during hydrogenation of variously substituted 3,4-dihydroisoquinolines and tried to interpret obtained results via means of computational chemistry.

